# Supplementary material for: Plasmodium falciparum hemozoin-associated biomolecules induce brain endothelial cell barrier disruption in an in vitro model of cerebral malaria
Source: mBio. 2026 Apr 20;17(5):e00313-26. doi: 10.1128/mbio.00313-26 (PMC13170363; doi:10.1128/mbio.00313-26)
Supplement: Caption — for Table S1. [file mbio.00313-26-s0003.docx]

**Table S1:** Complete dataset from mass spectrometric proteomic analysis of Hz-associated proteins.
